# Arthroscopic Management of Complex Knee Pathologies in a 45-Year-Old Male: A Case Report

**DOI:** 10.7759/cureus.64808

**Published:** 2024-07-18

**Authors:** Hatim Mohammed Alshareef, Ahmed A Elbarbari, Abdulaziz A Munshi, Yasser B Hennawi, Batool M Al-Otibi

**Affiliations:** 1 Orthopaedic Surgery, King Fahad Armed Forces Hospital, Jeddah, SAU; 2 Medicine, Umm Al-Qura University, Makkah, SAU

**Keywords:** case report, lateral meniscal cyst, knee pathology, chondromalacia patella, meniscus cyst

## Abstract

Knee disorders can present in various forms, often involving complex pathologies. The diagnosis and management of these conditions can be challenging, particularly in the absence of associated trauma. A 45-year-old male with a history of chronic right knee pain and clicking presented after failure of conservative treatment modalities. Imaging of the right knee identified multiple pathologies, including a ligament sprain, bone marrow edema, lateral maltracking of the patella, and advanced chondromalacia patella. Following these findings, the patient underwent arthroscopic surgery. A rare lateral meniscal cyst in the anterior horn was found during the surgery. Debridement, irrigation, and excision of the cyst were performed. Following the surgery, the patient experienced successful symptom resolution. Opting for arthroscopic surgery post other method failures can enhance patient outcomes.

## Introduction

The primary function of the knee, being one of the largest and most complex joints in the body, is to facilitate the flexion and extension of the leg. With its tibiofemoral and patellofemoral articulations, it is classified as a composite synovial joint [1]. However, the complexity of the knee also makes it prone to various injuries and disorders. Knee stability is mainly maintained by the anterior cruciate ligament (ACL), which restricts excessive tibial translation and rotation [2]. The medial and lateral menisci distribute weight across the knee, reduce friction, and protect against degenerative conditions [3]. The patella, a crucial part of the knee's extensor mechanism, can lead to degenerative conditions like chondromalacia patella when interacting with the femur [4]. Despite its crucial role in bodily movement and support, it is inherently vulnerable to a spectrum of injuries and disorders due to the intricate nature of its components and anatomical structure.

Meniscal cysts, commonly linked with meniscal tears, manifest as painful swellings on the knee joint's lateral side, often impeding mobility. Arthroscopic surgery is pivotal in treating these cysts, allowing for their decompression and simultaneous correction of the associated meniscal pathology [5]. Further, arthroscopy plays a vital role in managing chondral injuries, including chondromalacia patella, aiming to alleviate pain and improve joint function [6]. Moreover, arthroscopy is utilized in the evaluation and treatment of synovial diseases, such as synovitis, where synovectomy can be performed to remove inflamed tissue, thereby reducing pain and swelling [7]. Managing these pathologies through this approach ensures minimal disruption to the tissues and accelerates recovery, demonstrating the efficiency of arthroscopy in resolving complex knee disorders while minimizing patient discomfort. This study reports on a 45-year-old man with a lateral meniscal cyst 9x3 mm in size. That was associated with meniscal injury, ACL sprain and chondromalacia patella, which was treated with arthroscopy. The presence of a complex pathologic knee in our study adds a unique dimension to our paper.

## Case presentation

**Figure 1:**
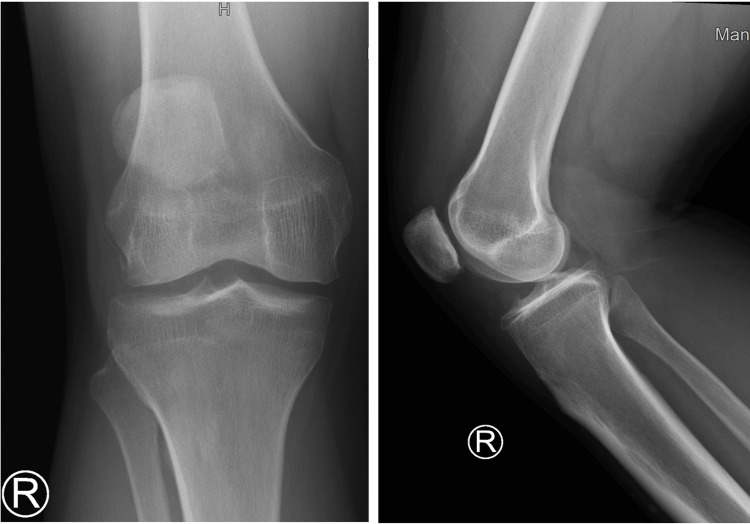
X-ray AP and lateral views of the knee joint show moderate osteoarthritis.

A 45-year-old male with a history of left ACL reconstruction but no known chronic medical conditions presented with complaints of chronic right knee pain and clicking for the past three years, without any recent traumatic episode. This absence of trauma history, combined with his persistent symptoms, identified this case as unique. Seeking medical advice, he consulted family medicine doctors who attempted various conservative measures including ice packs, analgesics, and physiotherapy strengthening exercises, all of which proved ineffective in managing his symptoms. Consequently, he was referred to the orthopedic team for re-evaluation. Upon examination, the patient exhibited anterior knee pain with tenderness along the lateral joint line. There was no knee effusion, and he had a full range of motion with pain experienced during full flexion. Mechanically stable in the varus-valgus stress test, the patient tested positive for the patellar grind test, while the Lachman and anterior drawer tests were negative. The McMurray test elicited lateral pain, and the Wilson test was positive. Furthermore, his neurovascular status remained intact, and X-rays showed moderate osteoarthritis (Figure 1).

**Figure 2:**
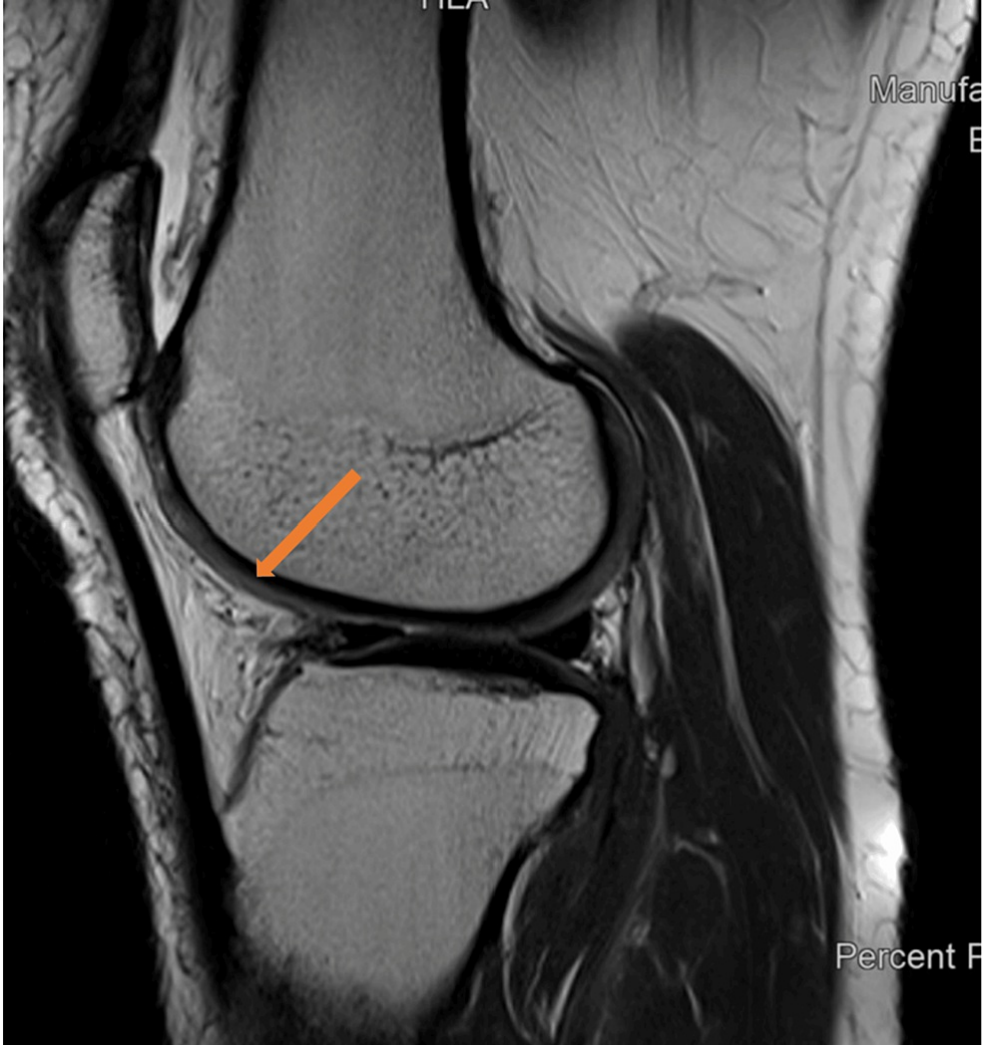
MRI sagittal view of knee joint showing degenerative changes and chondromalacia patella

Suspecting a meniscal injury and chondromalacia patella, an MRI of the right knee was done. The MRI, performed with a multi-sequential multiplanar protocol, showed compromised image quality due to motion artifacts. Despite this limitation, several significant findings have been identified. The anterior cruciate ligament (ACL) shows high signal intensity primarily at its tibial insertion. There is no definite disruption observed, which suggests the possibility of an ACL sprain. Additionally, there is bone marrow edema at the ACL tibial insertion, indicating an avulsion injury. However, no bony fragments are present in this area. Remarkably, there was also buckling of the posterior cruciate ligament (PCL), lateral malt racking of the patella, and advanced chondromalacia patella grade 4 findings that are rarely observed together, particularly in the absence of any recent trauma history (Figure 2). This combination of findings highlights the rarity of the case, offering a unique opportunity to explore the complexities of knee pathology.

**Figure 3:**
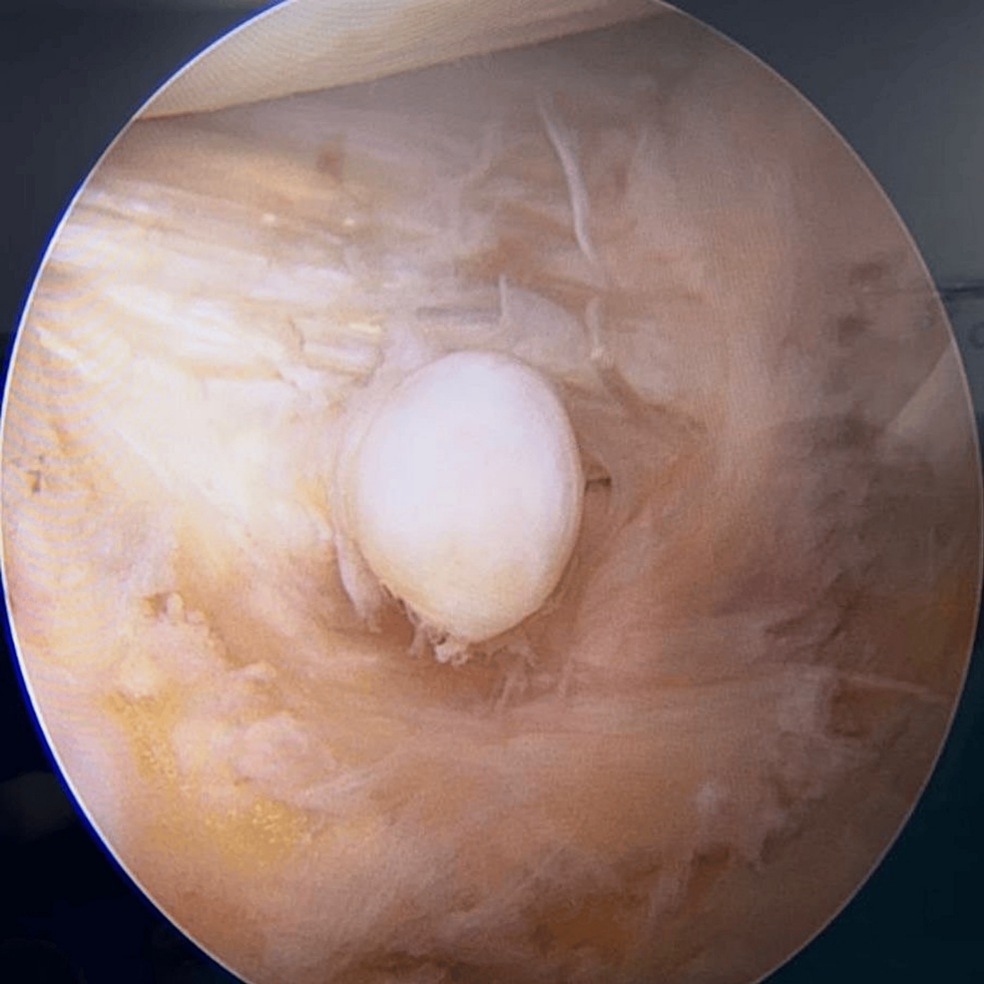
Arthroscopic view of the knee joint illustrating degenerative changes and meniscal cyst.

**Figure 4:**
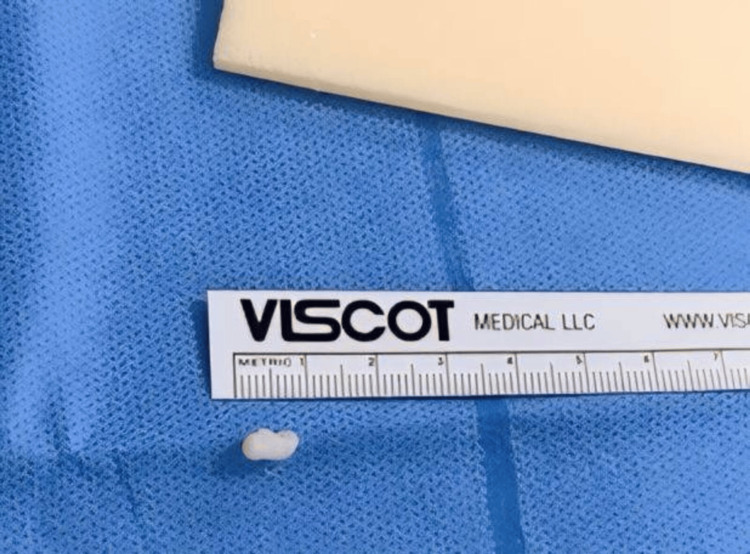
Excised meniscal cyst placed alongside the scale

Following the preparation, he underwent arthroscopic surgery of the right knee, which confirmed the MRI findings and additionally revealed chondromalacia patella with advanced degenerative changes predominantly affecting the lateral patellar facet, along with significant synovitis. Additionally, moderate osteoarthritic changes were observed in the medial tibiofemoral compartment, and a lateral meniscal cystic swelling was noted in the anterior horn, a rare finding that further emphasizes the uniqueness of this case (Figure 3). Debridement and irrigation were performed, along with the excision of the lateral meniscal cyst, which was found to be firm and measured 9x3 mm in size (Figure 4). The histology report described a myxoid collection of synovial epithelium with nonspecific inflammatory cells. The successful resolution of symptoms following debridement, cyst excision, and targeted intervention speaks to the efficacy of arthroscopic surgery in managing even the most complex and rare knee pathologies.

## Discussion

In the realm of knee pathologies, this case highlights an exceptional instance of complex knee pathology distinguished by a combination of factors such as advanced chondromalacia patella, meniscal degeneration, and the presence of a lateral meniscal cyst, all in the absence of trauma. Notably, the patient's history of persistent knee symptoms and a prior ACL reconstruction on the opposite knee deviates from the usual trauma-related knee injuries. For instance, Awwad et al. did a study in which the mean age was 24.1 years, with an average body mass index (BMI) of 29.2 kg/m^2^. Knee injuries were found to be the most common, with a rate of 616.7 injuries per 1000. Among knee injuries, medial cruciate ligament (MCL) and chondral/meniscal injuries were the most frequently occurring, accounting for 56.2% of all knee injuries [8]. This corresponds with our findings that MCL and chondral injuries such as chondromalacia patella are common among knee injuries, but distinguish them from cases in the elderly and those without a history of trauma. This case amplifies the clinical significance of considering a broader diagnostic spectrum in patients presenting with chronic knee pain, especially when traditional conservative treatments have failed.

Chondromalacia patellae (CP) is a condition identified by the softening, fraying, or ulceration of the cartilage located at the posterior of the patella, which is often accompanied by anterior knee pain. Studies have shown that approximately 50% of healthy individuals above the age of 20 years and nearly all individuals over the age of 50 years have patellar cartilage softening [9]. The prevalence of chondromalacia, and medial and lateral meniscus tears are significantly higher in ACL mucoid degeneration [10]. The probability of mucoid degeneration increases with age [10]. Although CP is relatively common, with a significant prevalence observed in individuals over 50 years of age, the advanced grade 4 chondromalacia patella observed in our patient, coupled with the absence of a precipitating traumatic event, underscores the uniqueness of this case. It also highlights the critical need for early and accurate diagnosis, given the progressive nature of the disease and its potential to significantly impair quality of life. Conservative treatment strategies for chondromalacia encompass non-pharmacological approaches such as patient education, self-care techniques, physical therapy, and weight reduction and cold therapy is a viable option. On the other hand, pharmacological interventions involve the use of nonsteroidal anti-inflammatory drugs (NSAIDs), acetaminophen, oral opioids, and duloxetine [11]. Our patient underwent these treatments before undergoing surgical procedures.

Arthroscopic debridement of localized articular cartilage lesions in the knee has demonstrated favorable outcomes in the short and medium term following surgery, particularly concerning functional enhancement. That is the reason we planned debridement. The utilization of arthroscopic debridement may be deemed appropriate as an initial approach for treating focal cartilage injuries, irrespective of the size and depth of the defect [12].

The occurrence of meniscal cysts adds another layer of complexity to our case. Meniscal cysts are relatively uncommon, with reported incidences ranging from 1-8% in both histologic and magnetic resonance imaging (MRI) studies [13]. While they may be indicative of a degenerative process or trauma, approximately 50% of these cysts are believed to be a result of trauma [14]. Until recently, lateral meniscal cysts were thought to be more prevalent compared to cysts of the medial meniscus. However, recent research conducted by authors using improved imaging methods and arthroscopic examination suggests that approximately 59-66% of cysts are found medially, with 34-41% occurring laterally [13,15]. The identification of a lateral meniscal cyst in our patient, in the context of an overall balanced distribution between medial and lateral cysts reported in recent literature, emphasizes the diagnostic and therapeutic challenges presented by these cysts. Cyst formation arises due to the accumulation of synovial fluid, infiltration of synovial cells, and proliferation of chondrocytes. The meniscal cysts in the study varied in size, ranging from 0.5 to 4 cm along the greatest diameter, with a median cyst size of 1.25 cm, but in our case, the cyst measured 0.9 cm in greatest diameter [16]. Horizontal tears often involve cysts located in the vascular zone and parameniscal region, making it necessary to strive for a minimally invasive approach in meniscectomy to prevent long-term complications like degenerative arthritis [17].

In terms of managing meniscal cysts, the primary surgical approaches involve arthroscopic repair of the meniscus in conjunction with cyst excision. The method to treat depends upon the extent of damage. The selection of surgical procedures for cases involving compression of the common peroneal nerve by a meniscal cyst is contingent upon the extent of nerve damage [18]. In a systemic review of eighteen studies comprising 753 patients following arthroscopic surgery for meniscal cysts, 79.3% of patients were able to resume their previous level of physical activity, while 85.7% experienced either complete resolution or minimal knee symptoms [19]. Since the neurovascular status of this patient was intact and there was no involvement of the peroneal nerve, the cyst was excised through an arthroscopy procedure.

## Conclusions

This particular case illustrates the significant impact of arthroscopic procedures in managing complicated knee pathologies, particularly emphasizing the rarity of such presentations without any prior trauma. By effectively utilizing arthroscopy, which includes precise debridement and cyst removal, this case highlights the success of minimally invasive surgery in treating complex conditions such as ACL sprains, meniscal tears, and advanced chondromalacia patella. The distinctiveness of the results stresses the need for advancements in arthroscopic techniques and patient care, promoting ongoing innovation in both surgical practices and rehabilitation strategies. This method not only enables a comprehensive approach to diagnosis and treatment but also establishes a model for further exploration into the management of uncommon and intricate knee disorders, demonstrating the evolving landscape of orthopedic surgery.

## References

[1] Hyland S, Sinkler MA, Varacallo M (2024). Anatomy, Bony Pelvis and Lower Limb: Popliteal Region. In: StatPearls [Internet].

[2] Ellison AE, Berg EE (1985). Embryology, anatomy, and function of the anterior cruciate ligament. Orthop Clin North Am.

[3] Mameri ES, Dasari SP, Fortier LM, Verdejo FG, Gursoy S, Yanke AB, Chahla J (2022). Review of meniscus anatomy and biomechanics. Curr Rev Musculoskelet Med.

[4] Habusta SF, Coffey R, Ponnarasu S, Mabrouk A, Griffin EE (2024). Chondromalacia patella. In: StatPearls [Internet].

[5] Kamimura T (2023). Arthroscopic intrameniscal decompression for the treatment of lateral meniscal cysts. Arthrosc Tech.

[6] Macmull S, Jaiswal PK, Bentley G, Skinner JA, Carrington RW, Briggs TW (2012). The role of autologous chondrocyte implantation in the treatment of symptomatic chondromalacia patellae. Int Orthop.

[7] Wengle LJ, Hauer TM, Chang JS, Theodoropoulos J (2021). Systematic arthroscopic treatment of synovial chondromatosis of the knee. Arthrosc Tech.

[8] Awwad GE, Coleman JH, Dunkley CJ, Dewar DC (2019). An analysis of knee injuries in rugby league: the experience at the Newcastle Knights professional rugby league team. Sports Med Open.

[9] Pascual-Garrido C, Slabaugh MA, L'Heureux DR, Friel NA, Cole BJ (2009). Recommendations and treatment outcomes for patellofemoral articular cartilage defects with autologous chondrocyte implantation: prospective evaluation at average 4-year follow-up. Am J Sports Med.

[10] Cilengir AH, Akkus OK, Baysan C, Uluc E, Cilengir N, Tosun O (2023). Ancillary findings in distinguishing between anterior cruciate ligament mucoid degeneration and sprain on MRI: a practical approach. Acta Radiol.

[11] Kuwabara A, Cinque M, Ray T, Sherman SL (2022). Treatment options for patellofemoral arthritis. Curr Rev Musculoskelet Med.

[12] Totlis T, Marín Fermín T, Kalifis G, Terzidis I, Maffulli N, Papakostas E (2021). Arthroscopic debridement for focal articular cartilage lesions of the knee: a systematic review. Surgeon.

[13] De Smet AA, Graf BK, del Rio AM (2011). Association of parameniscal cysts with underlying meniscal tears as identified on MRI and arthroscopy. AJR Am J Roentgenol.

[14] Ryu RK, Ting AJ (1993). Arthroscopic treatment of meniscal cysts. Arthroscopy.

[15] Anderson JJ, Connor GF, Helms CA (2010). New observations on meniscal cysts. Skeletal Radiol.

[16] Wu CC, Hsu YC, Chiu YC, Chang YC, Lee CH, Shen HC, Huang GS (2013). Parameniscal cyst formation in the knee is associated with meniscal tear size: an MRI study. Knee.

[17] Screpis D, Natali S, Piovan G, Iacono V, Magnanelli S, Farinelli L, Zorzi C (2021). Autologous platelet-rich fibrin matrix-augmented repair for parameniscal cysts: surgical technique. Arthrosc Tech.

[18] Zhi He L, Jiang J, Lu F, Teng F, Wu M, Xia Y (2022). Arthroscopic treatment of lateral meniscal cyst causing peroneal nerve injury. J Coll Physicians Surg Pak.

[19] Haratian A, Bolia IK, Hasan LK (2021). Arthroscopic management of meniscal cysts: a systematic review. Orthop Res Rev.

